# Superinvolution of the Uterus Following Trachelorraphy

**Published:** 1888-01

**Authors:** Virgil O. Hardon

**Affiliations:** Professor of Obstetrics and Diseases of Women and Children, Atlanta Medical College, Atlanta, Ga.


### Clinic Reports.

### SUPERINVOLUTION OF THE UTERUS FOLLOWING TRACHELORRAPHY.

BY VIRGIL O. HARDON, M. D., PROFESSOR OF OBSTETRICS AND DISEASES OF WOMEN AND CHILDREN, ATLANTA MEDICAL COLLEGE, ATLANTA, GA.

Two cases which have recently come under my observation have led me to believe that in exceptional instances the completion of the arrested process of involution which follows trachelorraphy may proceed beyond the physiological limits and thus induce a condition of atrophy of the uterus resembling the senile atrophy which normally occurs after the menopause. It is a well-known fact that such atrophy may occur in young, child-bearing women as a result of too frequent pregnancies. This condition was first recognized by Sir Jas. Y. Simpson, who gave to it the name of "superinvolution of the uterus." [a] Munde has made reference to it in a recent article,[b] although he fails, as do do Thomas and Emmet, to mention it in his text-book on gynæcology. Rockwell speaks of it and cites a case which was cured by the use of electricity. [c] Two years ago I reported a case in this Journal. [d] When this condition is present the uterus is small, approaching the infantile character, the cervix participates in the change, the vaginal portion becomes shorter and the os uteri diminishes in size. Menstruation becomes scanty and sometimes ceases altogether. Peculiar hysterical symptoms are at the same time developed, the so-called hystero-neuroses, such as melan-

---

[a] Clinical Lecture on Amenorrhoea, Medical Times and Gazette, 1861.

[b] American Journal of Obstetrics, December, 1885, p. 1254.

[c] American System of Gynæcology, Vol. I. p. 394.

[d] Atlanta Medical and Surgical Journal, Sept. 1885, p. 422.

cholia, persistent insomnia, neuralgia, especially pain in the cervical and lumbar regions of the spinal cord, mental depression, loss of appetite, indigestion and general weakness.

In the two cases in which I have seen this condition follow the operation of trachelorraphy, the laceration of the cervix was uncomplicated by any other lesion and the symptoms which called for the operation were due entirely to subinvolution. In both cases the repair of the cervix restored the patient to a condition of robust health, which continued for several months and was followed by a gradual development of nervous symptoms, suppression of menstruation and an atrophic condition of the uterus as shown by physical examination. The treatment of these symptoms was based upon a diagnosis of superinvolution of the uterus and consisted of frequent and long-continued intra-uterine faradization extending over a period of two months in one case and three months in the other. The results of this treatment were eminently satisfactory and served to confirm the diagnosis The womb was restored to its normal size, menstruation was re-established and the nervous symptoms entirely disappeared.

In searching the literature of laceration of the cervix and its treatment by trachelorraphy, I have been unable to find any allusion to superinvolution of the uterus as a possible sequel. Statistics which show the results of the operation after a length of time sufficient for the development of this condition, are exceedingly scanty. Van der Warker [a] has given the histories of 31 cases which he followed up for periods averaging 1½ years. In none of these do the results as given indicate superinvolution. In 19 cases in my own practice which I have been able to watch for longer periods than nine months, the two herein reported are the only ones which presented this train of symptoms.

CASE I. Mrs. H. B. L., white, married, aged 32, suffered laceration of the cervix in her third labor six years ago. Since that time has experienced bearing down sensations, backache, pain in loins, frequent micturition, leucorrhœa, menorrhagia, headache,

[a] American Journal of Obstetrics, July, 1883.

insomnia, neuralgia, mental depression and indigestion. Examination showed a bilateral laceration of the cervix, the womb in a state of subinvolution, three inches deep. June 7th, 1886, trachelorraphy was performed, perfect union followed and patient returned to her home. Three and a half months later she was seen by me and was then free from all her former symptoms, both local and general.

In March, 1887, nine months after the operation, the menses which had been normal since the operation, began to become scanty. At the same time there developed neuralgia of the head and back of the neck with pains up and down the spine. Vertigo, dimness of vision so that she was unable to read or write, insomnia, great mental depression with hysterical tendencies, loss of appetite and flesh, all followed, so that by midsummer she seemed a complete wreck.

On the 16th of August she entered my private infirmary for treatment. Examination showed the laceration to have been completely and permanent cured. The womb was small, being scarcely two inches deep, and felt dense and hard to the touch. The cervix was small and the canal with difficulty admitted a Simpson's uterine sound. No tenderness or hardness in the cellular tissue, no leucorrhœa and no local pain. The menses lasted only a few hours at each period and were pale and watery.

The patient was placed upon nourishing diet and a tonic of iron, quinine and strychnia. The faradic current of electricity was applied directly to the womb for half an hour on every second day. The positive pole was placed in the uterine cavity and the negative pole applied to the hypogastrium. This treatment was continued for three months. Under the stimulus of the electric current the womb gradually increased in size until it reached a depth of 2½ inches. The menses were restored to their normal color, quantity and duration. The nervous symptoms gradually disappeared and the patient recovered her full strength and health.

CASE 2.—Mrs. J. T. S., white, married, aged 27. Always well until the birth of her child two years ago. Since then has suffered from backache, pain in the pelvis, difficult and frequent micturition, painful defecation, nervous depression, insomnia and indigestion. The indigestion has become a very prominent feature and during the past year she has been under constant treatment for this symptom by various physicians without relief. Examination showed the womb to be enlarged, 3¼ inches deep, low in the pelvis and tender to the touch. Laceration of the cervix on the left side with eversion of the lips. February 26th, 1887, trachelorraphy was performed and perfect union followed. The relief of the symptoms was immediate. The persistent indigestion which had resisted all remedies was at once relieved and did not return. The local pains disappeared and the nervous symptoms subsided. The two menstrual periods following the operation were normal in all respects. In May the flow was very scanty, lasting only a day and a half, and in June did not appear at all. In other respects the patient's health remained good. Examination made July 1st showed the womb to be only two inches in depth and hard and dense to the touch. The laceration was entirely cured and there was no pain or hardness in any portion of the pelvic cellular tissue. Intra-uterine faradization was applied three times a week and daily for the week preceding the time of the menstrual period. The menses appeared for two days in July and three days in August. The electrical treatment was continued for two months during which time the womb gradually increased to a depth of 2⅜ inches and the menses became normal and have remained so ever since.

